# A Rapid Detection of Whole Wheat Gluten Quality by a Novel Chemometric Technique—GlutoPeak

**DOI:** 10.3390/foods11131927

**Published:** 2022-06-28

**Authors:** Secil Turksoy, Demet Onar

**Affiliations:** 1 Food Engineering Department, Hitit University, Corum 19030, Turkey; 2 Department of Food Engineering, Graduate School of Natural and Applied Sciences, Hitit University, Corum 19030, Turkey; demetonar@gmail.com

**Keywords:** whole wheat quality, gluten aggregation, GlutoPeak

## Abstract

The study aims to accurately detect gluten quality of whole wheat flour without a refining process by measuring gluten aggregation properties with a novel and non-destructive chemometric technique called GlutoPeak, coupled with principal component analyses (PCA) and hierarchical cluster analyses (HCA). For this purpose, whole wheat flour samples from 125 common bread wheat cultivars were analyzed for protein content (PC), wet gluten content (WGC), and Zeleny sedimentation value (SV). The correlations of GlutoPeak indices (peak maximum time, PMT; maximum torque, MT; torque 15 s before MT, AM; torque 15 s after MT) with other conventional wheat quality parameters were evaluated. Results indicated that MT had high correlations with WGC (*r* = 0.627, *p* < 0.05) and PC (*r* = 0.589, *p* < 0.05) while PC (*r* = 0.511, *p* < 0.05) and WGC (*r* = 0.566, *p* < 0.05) values had moderate correlations with the GlutoPeak PM index. Considering the effect of regions, the MT and PM GlutoPeak indices are powerful parameters to discriminate whole wheat flour samples by their gluten strengths. In conclusion, the GlutoPeak test can be a powerful and reliable tool for prediction of refined and unrefined wheat quality without being time-consuming.

## 1. Introduction

Gluten quality is the most important criteria both to characterize the flour performance and to decide its end use in the cereal industry [1]. The effect of gluten proteins on the structural, rheological, and textural properties of various wheat flour products have been controlled primarily by the main storage protein sub-fractions, monomeric gliadins, and polymeric glutenins [2]. Gluten quality, which should be in a balance between viscoelasticity and cohesiveness, is primarily controlled by genetic conditions. Therefore, evaluation of gluten quality has great importance for wheat breeding programs and the milling industry [1].

Widescale innovative analytical techniques based on chemical, electrophoretic, immunological, and spectroscopic factors have been developed and applied to determine and evaluate the gluten quality of wheat cultivars [2]. Empirical rheological measurements including dough mixing (Farinograph, Mixograph, Mixolab) and extension (Extensograph, Alveograph) have been used for years to evaluate gluten strength. Chemical test methods including hydrophilicity, the sodium dodecyl sulfate sedimentation test, the Zeleny sedimentation test, gluten index, water absorption capacity, and gliadin/glutenin ratio have some limitations such as repeatability, accuracy, variability from person to person, time consumption, large sample size, etc. These factors make the current techniques unsuitable for early-generation wheat breeding programs and the milling industry [1,3,4]. Particularly millers, breeders and bakers are in search of fast and reliable methods to predict gluten strength in a short time with limited sample contents [3].

GlutoPeak is a quick tool that can determine the gluten quality and fingerprint, and classify the wheat-based flours, measure the main mixing parameters (such as water absorption capacity, gluten aggregation and dough development time), characterize the gluten components, and predict the rheological behavior of different wheat flours [1,5,6,7]. The recently suggested shear-based device GlutoPeak provides a rapid evaluation for gluten quality of wheat flours with a small amount of sample. Aggregation behavior of gluten is measured by plotting a torque-time curve of solvent–sample mixture corresponding to applied mechanical force. This graph enables us to reach some important GlutoPeak indices, such as the peak maximum time, torque maximum, torque before maximum, and torque after maximum, which demonstrate significant correlation with conventional parameters [4,8].

In the present study, many unrefined wheat flour samples were evaluated by both conventional chemical quality tests used in cereal chain and a recently introduced chemometric GlutoPeak test method. Correlation between results obtained from both conventional and GlutoPeak measurements were examined. It is strongly believed that the results of this study will provide valuable information about the general chemical composition of selected whole wheat flour samples. The results enable us to classify the whole grain flour samples by their end-product functional properties at raw material receiving stage. Moreover, this study demonstrates the GlutoPeak usability as a fast and reliable evaluation method, particularly for millers and wheat breeders.

## 2. Materials and Methods

**Figure 1:**
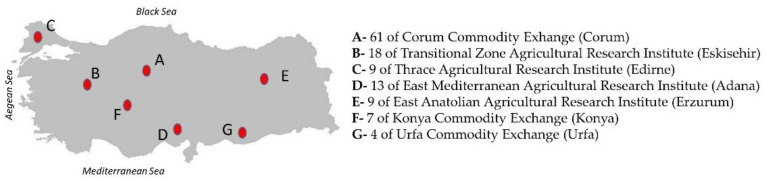
Regional distribution of wheat samples (*n* = 125).

A total of 125 winter wheat varieties harvested during the 2018–2019 growing period in rainfed conditions were obtained from different experimental farms located in Turkey (Adana, Çorum, Edirne, Erzurum, Eskişehir, Konya, Urfa) (Figure 1). Wheat samples (~2 kg) were milled on a Buhler laboratory mill (BreakMill SM 3, Brabender Ohg Duisburg, Germany) equipped with a 0.5 mm sieve after purging any impurities by sieving. Flour samples were kept in plastic bags and properly stored at refrigerator temperature (+4 °C) until experiments. All measurements were performed in duplicate. 

Moisture content (MC), ash content (AC), protein content (PC), wet gluten content (WGC), and Zeleny sedimentation value (SV) of whole wheat flour (WWF) samples (*n* = 125) were determined according to the AACC method 44-01, 08-01, 46-12, 38-10, and 56-61A, respectively [9].

The gluten aggregation properties of whole wheat flour samples were determined using the Brabender GlutoPeak device (Brabender GmbH&Co KG, Duisburg, Germany) as described by [6] with some modifications. For analysis, whole wheat flour (9 g, 14% mb) was dispersed in 9 g of 0.5 M CaCl_2_ solution at 35 °C by circulating water through the jacketed sample cup. The paddle was set to rotate at 2750 rpm and the test was performed for 5 min. All measurements were performed in duplicate, and the average of the results was used for further analysis. The primary evaluation parameters automatically provided by the instrument are peak maximum time (PMT, corresponding to the time of maximum torque, expressed in s), maximum torque (MT, corresponding to the maximum torque occurring due to optimum gluten aggregation, expressed in GlutoPeak Unit-GPU), torque 15 s before MT (AM, expressed in GlutoPeak Unit-GPU), and torque 15 s after MT (PM, expressed in GlutoPeak Unit-GPU).

All measurements (mean ± standart deviation) were performed in duplicate. Statistical analyses were conducted using one-way ANOVA (Minitab^®^19, 2020) at a significance level of 0.05. Tukey’s test was used to differentiate among the mean values (Table S1). Pearson’s correlation coefficients (*r*) were determined to analyze the relationship between the GlutoPeak indices and the commercial gluten quality parameters. For evaluating the sample discrimination, principal component analysis (PCA) and hierarchical cluster analyses (HCA) were also employed.

## 3. Results

**Figure 2:**
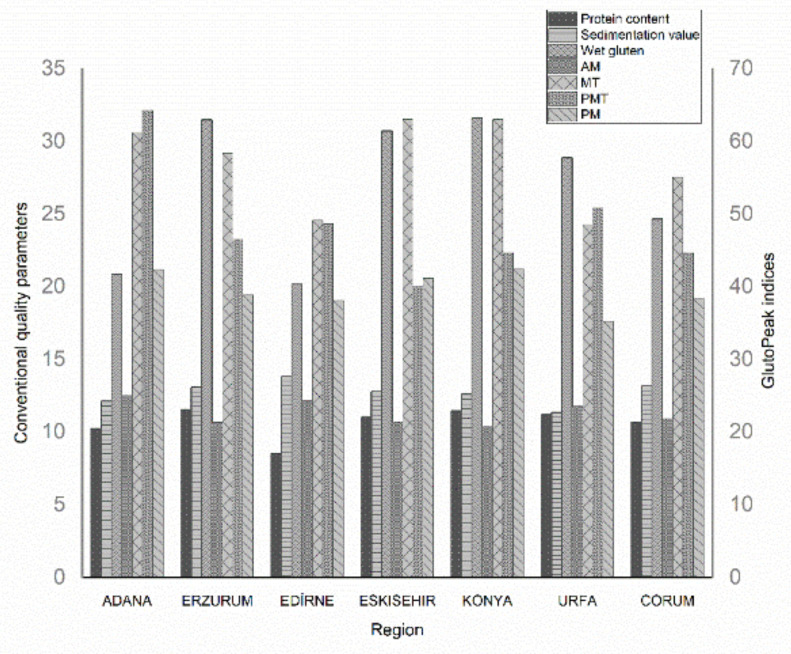
Distribution of average values of whole wheat flour samples due to different regions.

Conventional quality parameters and GlutoPeak indices of WWF samples (*n* = 125) are provided in Figure 2 and Table S1 (Supplementary Materials).

It was discovered that region and variety had significant effects on both conventional and GlutoPeak quality parameters of WWF samples (*p* < 0.05). Among the chemical parameters, ash contents of WWF samples changed the values between 1.4% and 2.1%. Ash content, one of the most important chemical quality parameters, determines the technological properties, mineral content, extraction rate, and nutritional labelling of flour samples [10]. Because the flour samples used in this study were obtained from whole grains (100% yield), the ACs were found above the limits determined by Turkish Alimentarius Codex legislations [11]. Protein contents of samples varied from a min 7.3% to a max 14% value due to the wheat variety, region, and environmental conditions. Flour samples obtained from hard wheat varieties have higher protein and gluten content with higher sedimentation values, as mentioned in literature [4,8]. Zeleny-SV changes depending on the climatic conditions and provides information about gluten quality, which is mostly determined by genetic factors [12]. Therefore, SV can be used for the classification of wheat flours since it is highly related to the protein content of flour [13]. WGC is another quality testing approach and provides information about the protein quality of wheat flour. In the study, wet gluten contents of whole wheat flour samples were discovered in the range from 7.5% to 48.2% with an average of ~26%. The ratio of wet gluten to protein content (WGC/PC) that demonstrates high correlation with baking characteristics has been considered an important indicator of WG production per protein unit of wheat flour [12,14]. A strong correlation were also found between the WGC/PC ratio, which ranged from 2.7 to 3.0, and optimal baking characteristics of gluten in the study, wherein 10 winter wheat were analyzed [15].

### Correlation of GlutoPeak Indices with Conventional Quality Parameters

The correlation coefficients (*r*) between conventional quality parameters and GlutoPeak indices are provided in Table 1.

Generally, a negative moderate correlation (*r* = −0.551, *p* < 0.05) was found between PMT and WGC among all samples, regardless of region. Since PMT provides information about gluten aggregation kinetics, the wheat varieties having stronger gluten with higher WGC indicated a high peak (MT) with a shorter peak time (PMT) [4,16]. MT had a positive moderate correlation with PC (*r* = 0.589, *p* < 0.05) and a strong correlation with WGC (*r* = 0.627, *p* < 0.05). PMT and MT values are crucial indices to discriminate flour samples as weak and strong due to their gluten qualities [17]. Results of a study from 37 commercial flour samples support the high correlation between GlutoPeak MT index and dough strength [18]. Therefore, flour samples from stronger wheat varieties with high PC and WGC demonstrated higher MT values. Among all samples, PM indicating the extent of gluten network destruction demonstrated a positive moderate correlation both with PC (*r* = 0.511, *p* < 0.05) and with WGC (*r* = 0.566, *p* < 0.05), as listed in Table 1. PM value is also an important criteria, although not as important as MT, in discriminating whole wheat flour samples due to their gluten qualities [8]. these two values demonstrated a relationship with dough extensibility due to having a positive correlation with high-molecular weight glutenin subunits [12].

The effect of region on the correlation between GlutoPeak indices and conventional quality parameters was also portrayed in Table 1. Among the seven regions, Adana, Konya, Edirne, and Erzurum demonstrated a negative strong correlation in descending order (*r* = −0.782, *r* = −0.747, *r* = −0.687, *r* = 0.659, *p* < 0.05) between PMT and WGC values of flours, while Corum demonstrated a negative moderate correlation (*r* = −0.511, *p* < 0.05). The MT GlutoPeak indices of flour samples from Edirne produced a very strong positive correlation with both PC (*r* = 0.890, *p* < 0.05) and WGC (*r* = 0.825, *p* < 0.05), which are important conventional quality parameters. Additionally, MT of flour samples from Eskisehir, Erzurum, and Corum had positive strong correlations both with PC (*r* = 0.736, *r* = 0.690, and *r* = 0.626, respectively, *p* < 0.05) and WGC (*r* = 0.652, *r* = 0.753, and *r* = 0.694, respectively, *p* < 0.05). These very strong and strong correlations have been supported by previous findings, suggesting that the MT value is a critical parameter to differentiate protein quality of whole wheat flour samples [8,12,17]. Among GlutoPeak indices, AM value provides a foresight about glutenin content which is responsible for the elasticity of the gluten network. Therefore, higher AM values are associated with higher degrees of crosslinking, increased dough elasticity, and gluten strength [19]. In this study, it was determined that AM value had a positive strong correlation with both PC (*r* = 0.626, *p* < 0.05) and WGC (*r* = 0.694, *p* < 0.05) in Corum, while it demonstrated a positive moderate correlation with PC (*r* = 0.529, *p* < 0.05) in Erzurum and with SV (*r* = 0.492, *p* < 0.05) in Edirne. Although PM value does not have as strong of a discrimination power as other GlutoPeak parameters, such as MT and PMT, the PM value has a strong correlation with the gluten strength of whole wheat flours [12]. PM value of flour samples from all regions demonstrated positive very strong (*r* = 0.908 for Edirne, *p* < 0.05), strong (*r* = 0.785 for Edirne, *r* = 0.768 for Eskisehir and *r* = 0.627 for Konya, *p* < 0.05), and moderate correlations (*r* = 0.573 for Corum, *p* < 0.05) with PC, except Adana. Only in the Urfa region, a negative very strong correlation (*r* = −0.850, *p* < 0.05) between PM and PC was observed. The flour samples from the Erzurum, Edirne, Eskisehir, and Corum regions produced very strong (*r* = 0.821 for Erzurum, *p* < 0.05) and strong correlations (*r* = 0.778 for Edirne, *r* = 0.744 for Eskisehir and *r* = 0.652 for Corum, *p* < 0.05) in descending order between GlutoPeak PM index and conventional WGC parameters. Overall, protein contents (which are primary determinants of wheat quality) of whole wheat flour samples have been significantly affected by environmental conditions (temperature, humidity, climatic changes, growing seasons etc.). Therefore, correlations between GlutoPeak and conventional properties of whole wheat flour samples from different regions (in rainfed conditions at about 21–24 °C) demonstrated a range from moderate to very strong correlations due to their changing protein contents and gluten qualities.

**Figure 3:**
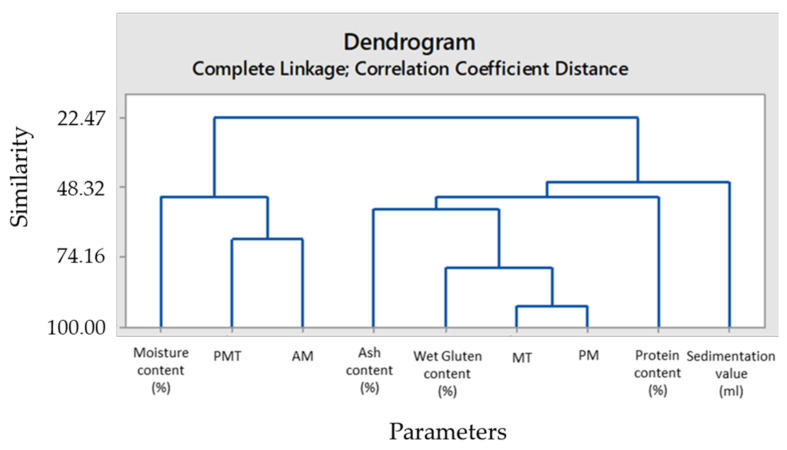
HCA dendogram of conventional gluten quality parameters versus GlutoPeak indices.

The results of PCA obtained from 9 variables and hierarchical clustering (HCA dendogram) of conventional and GlutoPeak gluten quality parameters were presented in Figure 3 and Table 2.

In Table 2, the eigenvalues of each variable and their ratios to explain the total change are reported both individually and cumulatively. In HCA dendogram (Figure 3), vertical lines indicate the distance between the clusters when they are combined, which means that longer branches suggest lesser similarity while shorter branches suggest higher similarity. The HCA dendogram highlighted that wet gluten content among traditional gluten quality parameters indicated the highest positive similarity (78%) with MT and PM GlutoPeak indices. The HCA dendogram had 95% specificity and 100% sensitivity.

## 4. Conclusions

In the present study, GlutoPeak and conventional test parameters of whole wheat flours from different regions of Turkey were compared. The results prove that it is possible to accurately predict some conventional quality indices of whole wheat flour samples in the GlutoPeak test. Significant correlations obtained from results enabled us to discriminate the whole wheat flours with varying gluten qualities. GlutoPeak indices, particularly MT and PM values, demonstrated strong correlations with protein content, wet gluten content, and Zeleny-sedimentation values of whole wheat flour samples. The results of this study have strongly indicated that GlutoPeak is a promising instrument for testing the gluten quality of both refined and whole wheat flours without being time consuming. Therefore, gluten quality of different wheat flours can be determined accurately and rapidly using the GlutoPeak test method, benefitting both wheat breeding programs and the flour milling industry.

## Figures and Tables

**Table 1:** General and regional correlation coefficients (*r*) between conventional and GlutoPeak parameters.

Region	Parameter	MC, %	AC, %	PC, %	SV, mL	WGC, %	PMT (GPU)	MT (GPU)	AM (GPU)
General	AC, %	0.124 *	1						
	PC, %	−0.056	0.314 *	1					
	SV, mL	−0.075	0.044	0.002	1				
	WGC, %	−0.195 *	0.363 *	0.780 *	−0.044	1			
	PMT	0.173	−0.211 *	−0.372 *	0.070	−0.551 *	1		
	MT	0.002	0.172 *	0.589 *	−0.072	0.627 *	−0.315 *	1	
	AM	0.042	−0.104	−0.179 *	−0.046	−0.184 *	0.356 *	0.005	1
	PM	−0.026	0.131	0.511 *	−0.007	0.566 *	−0.224 *	0.843 *	−0.007
Adana	AC, %	0.329	1						
	PC, %	−0.213	0.010	1					
	SV, mL	0.135	0.320	0.618 *	1				
	WGC, %	−0.348	−0.159	0.563 *	0.393 *	1			
	PMT	0.275	−0.165	−0.369	−0.427 *	−0.782 *	1		
	MT	−0.257	−0.662 *	−0.174	−0.398 *	−0.259	−0.079	1	
	AM	0.124	−0.349	−0.390 *	−0.581 *	−0.419 *	0.613 *	0.546 *	1
	PM	−0.170	−0.586 *	−0.057	−0.401 *	0.252	−0.083	0.932 *	0.618 *
Erzurum	AC, %	0.257	1						
	PC, %	0.247	0.753 *	1					
	SV, mL	−0.211	−0.072	0.287	1				
	WGC, %	0.430	0.483 *	0.893 *	0.329	1			
	PMT	−0.110	−0.114	−0.456	−0.081	−0.659 *	1		
	MT	0.416	0.391	0.690 *	−0.145	0.753 *	−0.499 *	1	
	AM	−0.096	0.585 *	0.529 *	0.347	0.299	−0.216	0.207	1
	PM	0.373	0.544 *	0.785 *	−0.086	0.821 *	−0.595 *	0.962 *	0.306
Edirne	AC, %	0.195	1						
	PC, %	−0.372	−0.007	1					
	SV, mL	−0.219	0.143	−0.305	1				
	WGC, %	−0.410 *	0.020	0.876 *	−0.497 *	1			
	PMT	0.235	0.156	−0.503 *	0.546 *	−0.687 *	1		
	MT	−0.349	−0.090	0.890 *	−0.264	0.825 *	−0.412 *	1	
	AM	0.104	0.224	−0.021	0.492 *	−0.334	0.638 *	−0.007	1
	PM	−0.288	0.040	0.908 *	−0.232	0.778 *	−0.386	0.926 *	0.214
Eskişehir	AC, %	−0.029	1						
	PC, %	−0.307	0.677 *	1					
	SV, mL	−0.340 *	−0.211	−0.02	1				
	WGC, %	−0.235	0.678 *	0.781 *	−0.048	1			
	PMT	−0.316	−0.239	−0.119	−0.071	−0.294	1		
	MT	−0.098	0.410 *	0.736 *	−0.239	0.652 *	0.041	1	
	AM	−0.180	0.094	0.343 *	−0.015	0.165	0.526 *	0.508 *	1
	PM	−0.133	0.421 *	0.768 *	0.019	0.744 *	−0.064	0.928 *	0.490 *
Corum	AC, %	0.122	1						
	PC, %	0.049	0.258 *	1					
	SV, mL	0.041	0.034	0.062	1				
	WGC, %	−0.135	0.274 *	0.757 *	−0.054	1			
	PMT	0.103	−0.080	−0.435 *	0.263 *	−0.511 *	1		
	MT	−0.201 *	0.122	0.626 *	0.088	0.694 *	−0.505 *	1	
	AM	0.042	−0.061	−0.163	−0.065	−0.135	0.222 *	−0.062	1
	PM	−0.191 *	0.055	0.573 *	0.113	0.652 *	−0.344 *	0.766 *	−0.200 *
Konya	AC, %	−0.018	1						
	PC, %	−0.725 *	−0.075	1					
	SV, mL	−0.554 *	0.246	0.088	1				
	WGC, %	−0.919 *	0.098	0.864 *	0.281	1			
	PMT	0.790 *	0.205	−0.743 *	−0.148	−0.747 *	1		
	MT	−0.485	0.092	0.528	0.505	0.370	−0.475	1	
	AM	0.325	−0.134	−0.210	0.105	−0.437	0.338	0.516	1
	PM	−0.591 *	0.091	0.627 *	0.403	0.520	−0.565 *	0.952 *	0.447
Urfa	AC, %	0.680	1						
	PC, %	0.880 *	0.943 *	1					
	SV, mL	−0.702	−0.872 *	−0.864 *	1				
	WGC, %	0.597	0.386	0.538	−0.278	1			
	PMT	−0.660	−0.135	−0.367	0.320	0.076	1		
	MT	0.362	−0.309	−0.050	0.053	−0.184	−0.893 *	1	
	AM	−0.541	−0.555	−0.576	0.633	0.316	0.736 *	−0.420	1
	PM	−0.597	−0.895 *	−0.850 *	0.704	−0.687	−0.160	0.527	0.153

*: Correlation is significant at 5% level. AC: ash content, PC: protein content, SV: Zeleny sedimentation value, WGC: wet gluten content, PMT: peak maximum time, MT: maximum torque, AM: torque 15 s before maximum torque, PM: torque 15 s after maximum torque, GPU: GlutoPeak Unit.

**Table 2:** Eigen values of the correlation matrix.

Eigenvalue 0.1449	2.7803	1.3299	1.1336	1.0553	0.9671	0.7812	0.5229	0.2848
Proportion 0.016	0.309	0.148	0.126	0.117	0.107	0.087	0.058	0.032
Cumulative 1.000	0.309	0.457	0.583	0.700	0.807	0.894	0.952	0.984

## Data Availability

The data presented in this study are available on request from the corresponding author. The data are not publicly available due to the privacy of research participants.

## Supplementary Materials

The following supporting information can be downloaded at: https://www.mdpi.com/article/10.3390/foods11131927/s1, Table S1: Chemical, physicochemical, and GlutoPeak indices of whole wheat flour samples (*n* = 125).

## References

[1] Chandi G.K., Seetharaman K. (2012). Optimization of Gluten Peak Tester: A Statistical Approach. J. Food Qual..

[2] Ooms N., Delcour J.A. (2019). How to Impact Gluten Protein Network Formation during Wheat Flour Dough Making. Curr. Opin. Food Sci..

[3] Malegori C., Grassi S., Ohm J.-B., Anderson J., Marti A. (2018). GlutoPeak Profile Analysis for Wheat Classification: Skipping the Refinement Process. J. Cereal Sci..

[4] Mecitoğlu Güçbilmez Ç., Şahin M., Göçmen Akçacık A., Aydoğan S., Demir B., Hamzaoğlu S., Gür S., Yakışır E. (2019). Evaluation of GlutoPeak Test for Prediction of Bread Wheat Flour Quality, Rheological Properties and Baking Performance. J. Cereal Sci..

[5] Marti A., Cecchini C., D’Egidio M.G., Dreisoerner J., Pagani M.A. (2014). Characterization of Durum Wheat Semolina by Means of a Rapid Shear-Based Method. Cereal Chem..

[6] Melnyk J.P., Dreisoerner J., Bonomi F., Marcone M.F., Seetharaman K. (2011). Effect of the Hofmeister Series on Gluten Aggregation Measured Using a High Shear-Based Technique. Food Res. Int..

[7] Wang J., Hou G.G., Liu T., Wang N., Bock J. (2018). GlutoPeak Method Improvement for Gluten Aggregation Measurement of Whole Wheat Flour. LWT Food Sci. Technol..

[8] Marti A., Ulrici A., Foca G., Quaglia L., Pagani M.A. (2015). Characterization of Common Wheat Flours (*Triticum aestivum* L.) through Multivariate Analysis of Conventional Rheological Parameters and Gluten Peak Test Indices. LWT Food Sci. Technol..

[9] (2003). AACC Approved Methods of the American Association of Cereal Chemists.

[10] Sezer B., Bilge G., Sanal T., Koksel H., Boyaci I.H. (2017). A Novel Method for Ash Analysis in Wheat Milling Fractions by Using Laser-Induced Breakdown Spectroscopy. J. Cereal Sci..

[11] Codex Alimentarius (2013). Turkish Food Codex Wheat Flour Announcement.

[12] Karaduman Y., Önder O., Sayaslan A., Aydın N. (2019). Utilisation of GlutoPeak Tester on Whole-Wheat Flour for Gluten Quality Assessment. Qual. Assur. Saf. Crops Foods.

[13] Hrušková M., Škodová V., Blažek J. (2004). Wheat Sedimentation Values and Falling Number. Czech J. Food Sci..

[14] Kaushik R., Kumar N., Sihag M.K., Ray A. (2015). Isolation, Characterization of Wheat Gluten and Its Regeneration Properties. J. Food Sci. Technol..

[15] Šimić G., Horvat D., Jurković Z., Drezner G., Novoselović D., Dvojković K. (2006). The Genotype Effect on the Ratio of Wet Gluten Content to Total Wheat Grain Protein. J. Cent. Eur. Agric..

[16] (2017). GlutoPeak Rapid Method for the Measurement of the Gluten Quality.

[17] Karaduman Y., Akin A., Türkölmez S., Tunca Z.Ş. (2015). Investigating the Usebility of GlutoPeak Tester for Evaluating Gluten Quality in Bread Wheat Breeding Programs. J. Field Crops Cent. Res. Inst..

[18] Huen J., Börsmann J., Matullat I., Böhm L., Stukenborg F., Heitmann M., Zannini E., Arendt E.K. (2018). Wheat Flour Quality Evaluation from the Baker’s Perspective: Comparative Assessment of 18 Analytical Methods. Eur. Food Res. Technol..

[19] Bouachra S., Begemann J., Aarab L., Hüsken A. (2017). Prediction of Bread Wheat Baking Quality Using an Optimized GlutoPeak^®^-Test Method. J. Cereal Sci..

