# Supplementary material for: A Rapid Detection of Whole Wheat Gluten Quality by a Novel Chemometric Technique—GlutoPeak
Source: Foods. 2022 Jun 28;11(13):1927. doi: 10.3390/foods11131927 (PMC9265637; doi:10.3390/foods11131927)
Supplement: Supplementary file 1 [file foods-11-01927-s001.zip › foods-1785993-supplementary.pdf]

Supplementary Material

Table S1. Chemical, physicochemical and GlutoPeak indices of whole wheat flour samples (n=125)

Region	Sample	Moisture Content (%)	Conventional Quality Indices				GlutoPeak Values			
			Ash content* (%)	Protein content* (%, N*5.7)	Zeleny sedimentation value** (mL)	Wet Gluten (%)	AM (GPU)	MT (GPU)	PMT (s)	PM (GPU)
ADANA	A-1	12.8±0.13^{defg}	1.52±0.01^{BC}	9.62±0.07^{B}	11.0±0.01^{BCD}	13.9±0.07^{B}	26.9±0.07^{E}	59.1±0.14^{C}	91.4±0.14^{L}	38.5±0.01^{B}
	A-2	12.6±0.12^{bcde}	1.40±0.04^{A}	9.63±0.05^{B}	10.5±0.71^{ABC}	16.2±0.42^{C}	26.5±0.07^{E}	59.5±0.64^{C}	90.2±0.21^{K}	41.1±0.07^{C}
	A-3	12.3±0.14^{b}	1.65±0.01^{DEF}	9.94±0.06^{C}	11.5±0.01^{D}	17.9±0.57^{D}	26.6±0.07^{E}	67.5±0.07^{G}	79.9±0.14^{I}	44.4±0.21^{D}
	A-4	13.1±0.04^{g}	1.91±0.01^{G}	10.31±0.03^{D}	14.8±0.35^{G}	20.9±0.57^{F}	14.5±0.01^{A}	41.5±0.71^{B}	38.1±0.07^{A}	33.1±0.14^{A}
	A-5	12.9±0.05^{efg}	1.58±0.01^{CDE}	9.81±0.06^{C}	10.0±0.01^{A}	12.1±0.57^{A}	32.0±1.41^{F}	64.0±0.01^{F}	77.7±0.28^{H}	46.5±0.01^{E}
	A-6	12.7±0.21^{bcdef}	1.66±0.02^{DEF}	10.4±0.02^{D}	10.8±0.35^{ABCD}	19.9±0.92^{EF}	34.5±0.01^{G}	64.5±0.01^{F}	81.3±0.35^{J}	46.7±0.21^{E}
	A-7	12.7±0.37^{cdef}	1.52±1.16^{BC}	10.76±0.04^{EF}	13.3±0.35^{F}	23.5±0.71^{G}	24.0±1.41^{CD}	73.2±0.42^{I}	50.3±0.35^{D}	47.0±1.41^{E}
	A-8	12.9±0.07^{efg}	1.72±0.01^{F}	11.35±0.04^{G}	15.0±0.01^{G}	19.3±0.49^{E}	20.5±1.41^{B}	38.4±0.14^{A}	77.1±0.14^{G}	32.0±1.41^{A}
	A-9	10.7±0.04^{a}	1.56±0.01^{CD}	10.63±0.13^{E}	11.3±0.35^{CD}	26.1±0.49^{H}	20.0±1.41^{B}	61.4±0.21^{D}	40.1±0.14^{B}	41.0±1.41^{C}
	A-10	12.9±0.08^{efg}	1.55±0.02^{CD}	10.34±0.08^{D}	10.3±0.35^{AB}	27.1±0.57^{HI}	22.6±0.07^{C}	64.0±0.01^{F}	55.0±0.01^{E}	44.0±0.01^{D}
	A-11	13.0±0.03^{fg}	1.68±0.01^{EF}	8.90±0.08^{A}	11.3±0.35^{CD}	19.4±0.42^{E}	27.5±0.07^{E}	63.0±0.01^{E}	59.0±0.01^{F}	41.5±0.01^{C}
	A-12	12.4±0.21^{bcd}	1.50±0.01^{ABC}	10.88±0.10^{F}	15.8±0.35^{H}	28.4±0.99^{I}	24.5±0.07^{D}	67.1±0.14^{G}	50.1±0.07^{D}	44.4±0.21^{D}
	A-13	12.4±0.15^{bc}	1.42±0.01^{AB}	10.66±0.08^{E}	12.3±0.35^{E}	26.6±0.57^{H}	25.6±0.14^{D}	72.5±0.07^{H}	45.6±0.14^{C}	51.2±0.28^{F}
ERZURUM	ER-1	10.8±0.39^{a}	1.44±0.01^{A}	11.87±0.03^{D}	17.3±0.35^{E}	37.1±1.70^{F}	21.6±0.14^{BC}	68.5±0.07^{F}	41.0±1.41^{C}	43.0±1.41^{E}
	ER-2	12.5±0.38^{d}	1.78±0.01^{C}	12.68±0.01^{E}	13.5±0.71^{D}	34.6±1.91^{DE}	21.0±1.41^{B}	63.0±0.41^{E}	62.0±0.40^{F}	38.0±1.41^{D}
	ER-3	13.4±0.06^{e}	1.87±0.01^{D}	13.48±0.06^{G}	12.8±0.35^{CD}	48.2±0.28^{G}	21.2±0.28^{BC}	81.5±0.07^{H}	30.0±1.41^{A}	53.6±0.14^{F}
	ER-4	11.2±0.06^{abc}	1.85±0.01^{D}	11.81±0.03^{D}	11.3±0.35^{AB}	26.9±0.28^{B}	23.3±0.35^{CD}	66.6±0.14^{F}	56.1±0.14^{E}	42.1±0.07^{E}
	ER-5	11.5±0.12^{bc}	1.50±0.06^{B}	7.88±0.05^{A}	11.5±0.71^{AB}	13.1±0.28^{A}	19.0±1.41^{A}	33.6±0.07^{A}	69.0±0.01^{G}	24.1±0.14^{A}
	ER-6	10.9±0.15^{a}	1.93±0.01^{E}	12.74±0.04^{E}	16.8±0.35^{E}	33.0±0.71^{D}	23.7±0.21^{D}	36.0±0.41^{B}	45.9±0.21^{D}	31.2±0.28^{B}
	ER-7	11.1±0.08^{ab}	1.97±0.02^{E}	13.32±0.04^{F}	10.8±0.35^{A}	36.6±0.07^{EF}	21.6±0.14^{BC}	77.3±0.35^{G}	36.0±1.41^{B}	52.6±0.07^{F}
	ER-8	11.6±0.08^{c}	1.47±0.01^{AB}	9.66±0.03^{B}	11.5±0.71^{ABC}	24.9±0.14^{B}	21.6±0.07^{BC}	51.0±1.41^{D}	34.0±1.41^{B}	33.0±0.01^{C}
	ER-9	10.9±0.06^{a}	1.46±0.01^{AB}	10.67±0.06^{C}	12.5±0.71^{BCD}	29.0±0.14^{C}	19.0±1.41^{A}	48.1±0.14^{C}	44.4±0.14^{D}	32.2±0.21^{BC}
EDİRNE	ED-1	11.2±0.21^{cd}	1.85±0.01^{F}	8.66±0.01^{E}	15.8±0.35^{FG}	18.1±0.14^{C}	29.0±1.41^{I}	50.3±0.14^{G}	69.0±0.01^{H}	39.0±1.41^{CD}
	ED-2	11.1±0.11^{c}	1.65±0.01^{BC}	7.31±0.01^{A}	16.3±0.35^{G}	14.3±0.57^{B}	24.6±0.07^{E}	39.6±0.14^{B}	57.0±1.41^{G}	32.1±0.07^{A}
	ED-3	10.4±0.06^{b}	1.65±0.01^{BC}	8.53±0.01^{D}	16.0±0.01^{G}	18.9±0.71^{C}	25.2±0.14^{EF}	53.7±0.21^{H}	52.1±0.14^{F}	39.5±0.07^{D}
	ED-4	12.0±0.04^{gh}	1.64±0.01^{B}	7.26±0.03^{A}	16.0±0.71^{G}	7.5±0.21^{A}	27.1±0.14^{G}	35.9±0.07^{A}	80.1±0.14^{I}	31.0±0.14^{A}
	ED-5	11.5±0.07^{ef}	1.73±0.01^{D}	8.65±0.10^{E}	13.8±0.35^{D}	23.8±0.28^{E}	19.4±0.07^{C}	56.0±1.41^{I}	31.0±0.01^{A}	39.1±0.07^{CD}
	ED-6	11.8±0.10^{fg}	1.73±0.03^{D}	8.69±0.01^{E}	9.3±0.35^{A}	26.4±0.57^{F}	16.8±0.01^{A}	48.3±0.14^{F}	36.2±0.28^{B}	36.5±0.07^{B}
	ED-7	9.9±0.11^{a}	1.68±0.01^{C}	9.57±0.04^{G}	14.3±0.35^{DE}	27.6±0.85^{G}	27.6±0.14^{GH}	58.1±0.07^{J}	39.1±0.07^{C}	44.3±0.07^{E}
	ED-8	10.5±0.25^{b}	1.52±0.01^{A}	8.86±0.01^{F}	12.3±0.35^{C}	22.1±0.28^{D}	18.1±0.14^{B}	50.0±1.41^{G}	38.0±1.41^{C}	36.0±1.41^{B}
	ED-9	11.3±0.26^{cde}	1.68±0.03^{C}	8.01±0.04^{C}	13.8±0.30^{D}	18.7±0.01^{C}	21.0±1.41^{D}	42.5±0.14^{C}	38.6±0.07^{C}	36.6±0.14^{B}
	ED-10	12.4±0.04^{i}	1.68±0.01^{C}	8.65±0.03^{E}	15.0±0.07^{EF}	14.1±0.57^{B}	28.5±0.07^{H}	45.6±0.07^{E}	48.1±0.14^{F}	38.8±0.01^{CD}
	ED-11	11.2±0.04^{cd}	1.76±0.01^{E}	8.81±0.06^{F}	14.8±0.05^{E}	24.0±0.42^{E}	24.6±0.14^{E}	44.2±0.28^{D}	46.5±0.01^{D}	37.6±0.07^{B}
	ED-12	11.4±0.14^{de}	1.62±0.01^{B}	10.05±0.02^{H}	11.8±0.30^{C}	32.2±0.28^{H}	26.4±0.14^{FG}	73.3±0.14^{K}	46.1±0.14^{D}	49.0±1.41^{F}
	ED-13	12.10±0.05^{h}	1.68±0.01^{C}	7.73±0.01^{B}	10.8±0.35^{B}	14.8±0.35^{B}	27.6±0.14^{GH}	41.3±0.14^{C}	51.3±0.35^{F}	36.2±0.21^{B}

*Values are calculated as dry basis. **Values are calculated as 14% moisture content.Values in the same column with different superscripts indicate a statistically significant difference ($p<0.05$). AM: torque 15s before maximum torque, MT: maximum torque, PMT: peak maximum time, PM: torque 15s after maximum torque, GPU: GlutoPeak Unit

Supplementary Material

Table S1. Continue

Region	Sample	Moisture Content (%)	Ash content* (%)	Protein content* (%, N*5.7)	Zeleny sedimentation value** (mL)	Wet Gluten (%)	AM (GPU)	MT (GPU)	PMT (s)	PM (GPU)
ESKİŞEHİR	ES-1	10.1±0.01a	1.75±0.04^{BC}	10.89±0.04^{F}	14.8±0.35^{HIJ}	33.3±0.01^{G}	23.2±0.25^{F}	60.6±0.18^{EF}	51.6±0.14^{H}	41.0±0.07^{D}
	ES-2	12.6±0.12^{hi}	2.00±0.05^{H}	11.86±0.04^{H}	11.8±0.35^{CD}	36.2±0.57^{H}	24.0±0.01^{F}	77.5±0.07^{J}	52.5±0.07^{H}	49.2±0.28^{H}
	ES-3	12.8±0.12^{i}	1.88±0.02^{F}	10.07±0.05^{D}	11.0±0.71^{ABC}	30.2±0.21^{EF}	20.5±0.71^{BC}	57.6±0.14^{D}	29.0±1.41^{A}	37.0±1.41^{C}
	ES-4	12.0±0.04^{e}	1.89±0.01^{F}	10.84±0.01^{F}	15.5±0.70^{IJ}	30.8±0.14^{F}	19.3±0.35^{AB}	42.0±1.41^{A}	36.0±1.41^{CDE}	34.0±1.41^{A}
	ES-5	12.3±0.28^{fg}	1.95±0.02^{G}	10.75±0.03^{E}	15.8±0.35^{J}	29.6±0.49^{DE}	21.2±0.28^{CDE}	59.0±1.41^{DE}	34.5±1.41^{BCDE}	42.0±1.41^{DE}
	ES-6	10.0±0.01^{a}	1.95±0.01^{G}	12.99±0.02^{K}	15.5±0.71^{IJ}	38.0±0.28^{I}	25.7±0.28^{G}	81.4±0.21^{K}	38.4±0.14^{EF}	52.0±1.41^{I}
	ES-7	12.5±0.13^{gh}	1.74±0.01^{C}	9.82±0.04^{C}	11.0±0.05^{ABC}	26.6±0.78^{C}	18.3±0.35^{A}	54.0±1.41^{C}	30.8±0.35^{AB}	35.3±0.35^{AB}
	ES-8	12.0±0.14^{e}	2.12±0.01^{J}	11.91±0.07^{I}	12.8±0.35^{EF}	33.4±0.78^{G}	20.8±0.35^{CD}	68.5±0.07^{H}	35.6±0.14^{CDE}	43.2±0.28^{EF}
	ES-9	12.1±0.08^{ef}	2.12±0.03^{J}	12.29±0.04^{J}	10.3±0.03^{AB}	36.7±0.49^{H}	21.5±0.01^{CDE}	72.0±1.41^{I}	33.5±0.07^{BCD}	44.5±0.64^{F}
	ES-10	12.2±0.05^{ef}	1.79±0.01^{CD}	10.85±0.14^{F}	11.3±0.35^{BC}	30.9±0.64^{F}	19.0±1.41^{A}	66.0±1.41^{G}	42.1±0.14^{FG}	42.0±0.01^{DE}
	ES-11	11.7±0.14^{d}	1.79±0.01^{CD}	12.22±0.19^{J}	13.8±0.30^{FGH}	30.8±0.85^{F}	18.0±1.41^{A}	66.6±0.07^{G}	32.4±0.93^{ABC}	43.3±0.35^{EF}
	ES-12	11.1±0.06^{b}	2.04±0.01^{I}	11.62±0.01^{G}	10.0±0.09^{A}	37.3±0.21^{HI}	19.0±1.41^{A}	65.7±0.28^{G}	37.5±0.01^{DE}	41.3±0.42^{D}
	ES-13	13.0±0.06^{j}	1.75±0.01^{BC}	10.65±0.06^{E}	13.8±0.35^{FGH}	32.4±0.49^{G}	24.2±0.21^{F}	70.5±0.07^{I}	32.4±0.21^{ABC}	47.1±0.14^{G}
	ES-14	11.4±0.08^{c}	1.90±0.01^{F}	10.87±0.01^{F}	13.5±0.71^{FG}	26.2±0.07^{C}	22.1±0.14^{DE}	52.1±0.14^{B}	44.5±0.01^{G}	36.3±0.35^{BC}
	ES-15	12.3±0.07^{fgh}	1.87±0.01^{EF}	11.76±0.01^{H}	10.0±0.01^{A}	26.9±0.64^{C}	28.5±0.01^{H}	71.1±0.07^{I}	53.1±0.14^{H}	42.1±0.07^{DE}
	ES-16	12.0±0.06^{e}	1.83±0.01^{DE}	10.66±0.06^{E}	13.0±0.05^{EF}	28.9±0.64^{D}	19.1±0.07^{A}	61.7±0.28^{F}	32.6±0.14^{ABC}	42.0±0.01^{DE}
	ES-17	12.0±0.05^{e}	1.72±0.01^{AB}	9.67±0.01^{B}	14.5±0.07^{GHI}	23.9±0.49^{B}	18.3±0.35^{A}	54.3±0.35^{C}	43.6±0.07^{G}	33.7±0.28^{A}
	ES-18	11.4±0.12^{c}	1.68±0.03^{A}	9.25±0.01^{A}	12.3±0.35^{DE}	20.9±0.28^{A}	22.5±0.07^{E}	54.1±0.14^{C}	60.0±0.41^{I}	34.9±0.14^{AB}
KONYA	K-1	11.1±0.09^{d}	1.41±0.02^{A}	10.93±0.04^{B}	11.8±0.35^{A}	26.3±0.07^{A}	23.6±0.07^{C}	60.4±0.14^{C}	52.3±0.35^{E}	41.2±0.28^{C}
	K-2	11.7±0.11^{e}	1.85±0.02^{E}	10.93±0.06^{B}	11.8±0.35^{A}	26.4±0.64^{A}	20.2±0.25^{B}	55.6±0.14^{A}	60.4±0.14^{F}	36.1±0.14^{A}
	K-3	10.1±0.09^{bc}	1.66±0.02^{C}	12.40±0.02^{D}	10.3±0.35^{A}	37.6±0.78^{D}	17.7±0.21^{A}	57.8±0.11^{B}	34.0±1.41^{A}	41.4±0.14^{C}
	K-4	10.3±0.08^{c}	1.93±0.01^{F}	10.50±0.04^{A}	13.8±1.06^{CD}	30.9±0.35^{B}	21.1±0.14^{B}	62.4±0.49^{D}	50.2±0.21^{D}	43.1±0.07^{D}
	K-5	9.9±0.12^{b}	1.57±0.01^{B}	11.10±0.08^{C}	13.8±1.06^{CD}	32.2±0.28^{C}	18.0±0.41^{A}	58.3±0.35^{B}	39.5±0.01^{B}	37.8±0.35^{B}
	K-6	11.1±0.08^{d}	1.70±0.02^{D}	11.16±0.07^{C}	12.3±0.35^{BC}	25.3±0.21^{A}	23.3±0.35^{C}	71.0±1.41^{E}	42.0±1.41^{C}	46.2±0.21^{E}
	K-7	8.8±0.12^{a}	1.73±0.01^{D}	13.29±0.08^{E}	14.5±0.71^{D}	42.6±0.42^{E}	21.5±0.07^{B}	75.7±0.28^{F}	34.3±0.35^{A}	51.3±0.35^{F}
URFA	SU-1	9.4±0.08^{a}	1.46±0.01^{A}	10.45±0.08^{A}	11.8±0.35^{B}	25.4±0.14^{A}	21.0±0.41^{B}	59.0±1.41^{C}	42.5±0.01^{B}	39.2±0.28^{D}
	SU-2	9.6±0.13^{a}	1.66±0.01^{C}	11.42±0.07^{C}	11.0±0.01^{AB}	26.8±0.78^{A}	18.0±1.41^{B}	39.3±0.35^{A}	58.2±0.28^{C}	34.0±0.07^{B}
	SU-3	9.4±0.08^{a}	1.52±0.01^{B}	10.77±0.01^{B}	11.8±0.35^{B}	31.4±0.71^{B}	41.0±0.30^{C}	41.7±0.28^{A}	63.9±0.14^{D}	35.0±0.01^{C}
	SU-4	11.8±0.06^{b}	1.69±0.01^{D}	12.26±0.03^{D}	10.8±0.35^{A}	31.9±0.14^{B}	14.3±0.35^{A}	54.0±1.41^{B}	38.6±0.07^{A}	32.9±0.21^{A}
CORUM	C-1	13.2±0.01$^{\gamma\delta\tau}$	1.65±0.02^{UVWXYZ}	11.04±0.04^{XYZabcdefghijkl}	10.3±0.35^{BCDE}	25.1±0.14^{PQRSTUVWX}	25.4±0.21^{klmnop}	43.0±1.41^{IJK}	59.1±0.14^{vw}	39.0±1.41^{WX}
	C-2	12.6±0.25^{stuvwyz}	1.81±0.01^{pqrstu}	10.86±0.12^{UVWXYZabcdefg}	10.3±0.35^{BCDE}	27.2±0.28^{STUVWXYZabcde}	20.6±0.14^{OPQRSTUVWX}	43.5±0.07^{JKL}	33.6±0.07^{HIJK}	37.6±0.07^{RSTUV}
	C-3	11.2±0.02^{PQRSYUVWY}	1.72±0.01^{bcdefghijk}	9.18±0.02^{GHIJKL}	10.3±0.35^{BCDE}	14.1±0.42^{CDE}	31.5±0.01^{u}	35.1±0.14^{DE}	81.5±0.01$^{\gamma}$	32.5±1.41^{GHIJ}
	C-4	11.2±0.02^{PQRSYUVWY}	1.76±0.05^{hijklmnop}	9.50±0.13^{HIJKLMN}	16.3±0.35^{YZab}	18.1±1.27^{GHIJ}	22.0±1.41^{UVWXYZabcdef}	42.9±0.14^{IJK}	43.0±1.41^{abcdef}	33.5±0.07^{IJK}
	C-5	11.5±0.62^{VWYXZabc}	1.67±0.04^{XYZabcd}	9.80±0.14^{KLMNOPQR}	14.3±0.35^{QRSTU}	26.6±0.14^{RSTUVWXYZabc}	23.3±0.35^{Zabcdefghijk}	61.2±0.28^{hij}	53.0±1.41^{pqr}	39.0±1.41^{WX}
	C-6	10.8±0.01^{IJKLMNOP}	1.54±0.02^{JKLMNOP}	10.70±0.7^{RSTUVWXYZabc}	14.5±0.71^{RSTUV}	27.1±0.49^{RSTUVWXYZabcd}	22.4±0.57^{WXYZabcdefgh}	67.0±0.01^{rst}	36.5±0.01^{LMNOP}	41.2±0.28^{Zab}
	C-7	10.5±0.07^{EFGHIJK}	1.60±0.03^{QRSTUVW}	9.02±0.16^{FGHIJK}	16.0±0.01^{XYZab}	19.1±0.01^{HIJK}	22.2±0.28^{VWXYZabcdefg}	51.2±0.21^{OP}	51.0±1.41^{nop}	36.1±0.07^{NOP}
	C-8	11.2±0.07^{QRSTUVWYX}	1.40±0.01^{BC}	11.39±0.04^{abcdefghijklmno}	14.8±0.35^{STUVW}	28.6±0.64^{WXYZabcdefgh}	17.9±0.14^{FGHIJKLM}	54.0±0.01^{RST}	27.5±0.07^{A}	38.7±0.42^{VWX}
	C-9	13.3±0.04$^{\delta\tau}$	2.00±0.04$^{\delta\tau}$	11.75±0.01^{ghijklmnopqr}	15.0±0.01^{TUVWX}	24.5±0.21^{OPQRSTU}	17.5±0.01^{EFGHIJKL}	55.6±0.07^{VWX}	45.5±0.01^{fghi}	39.0±1.41^{WX}
	C-10	12.5±0.04^{rstuvwy}	1.95±0.01$^{\alpha\beta\gamma\delta}$	10.93±0.59^{VWXYZabcdefghij}	14.0±0.01^{PQRST}	21.7±0.42^{KLMNOP}	31.4±7.21^{u}	44.1±0.14^{KL}	39.6±0.14^{QRSTUVWX}	36.8±0.35^{PQRST}

*Values are calculated as dry basis. **Values are calculated as 14% moisture content.Values in the same column with different superscripts indicate a statistically significant difference ($p<0.05$). AM: torque 15s before maximum torque, MT: maximum torque, PMT: peak maximum time, PM: torque 15s after maximum torque, GPU: GlutoPeak Unit

Supplementary Material

Table S1. Continue

Region	Sample	Moisture Content (%)	Ash content* (%)	Protein content* (%, N*5.7)	Zeleny sedimentation value** (mL)	Wet Gluten (%)	AM (GPU)	MT (GPU)	PMT (s)	PM (GPU)
CORUM	C-11	11.7±0.22^{Zabcdefgh}	1.80±0.01^{opqrstu}	9.97±0.01^{LMNOPQRSTU}	15.3±0.35^{UVWXY}	24.9±0.42^{PQRSTUVW}	22.2±0.28^{VWXYZabcdefg}	57.1±0.14^{YZab}	41.4±0.14^{WXYZab}	32.5±0.01^{GHIJ}
	C-12	11.0±0.06^{LMNOPQRS}	1.58±0.02^{NOPQRST}	9.29±0.01^{GHIJKLM}	13.3±1.06^{MNOPQ}	17.2±0.42^{EFGHI}	23.1±0.07^{YZabcdefghij}	51.9±0.14^{PQ}	52.1±0.07^{opq}	36.5±0.07^{OPQRS}
	C-13	12.0±0.01^{fghijklmn}	1.66±0.07^{WXYZabc}	10.58±0.06^{QRSTUVWXYZab}	10.0±0.01^{BCD}	29.8±0.64^{abcdefghijkl}	21.7±0.28^{TUVWXYZabcdef}	61.2±0.21^{hij}	42.4±0.14Y^{Zabcde}	43.1±0.14^{defgh}
	C-14	10.2±0.12^{ABCDE}	1.79±0.01^{nopqrstu}	13.00±0.06^{VW}	10.8±0.35^{CDEFG}	35.0±0.57^{opqrst}	23.0±1.41^{YZabcdefghij}	65.1±0.07^{nop}	40.5±0.01^{TUVWXYZa}	44.1±0.0^{fghi}
	C-15	11.9±0.08^{efghijklm}	1.62±0.01^{STUVWXY}	8.32±0.11^{BCDEF}	15.8±0.35^{WXYZa}	14.9±0.21^{CDEFG}	28.0±1.41st	36.1±0.07^{EF}	81.5±0.01$^{\gamma}$	32.6±0.14^{HIJ}
	C-16	11.7±0.03^{Zabcdefgh}	1.70±0.01^{Zabcdefghi}	15.19±3.82^{y}	17.3±0.35^{cd}	33.3±0.35^{lmnopq}	23.4±0.21^{abcdefghijkl}	68.5±0.01^{uv}	43.5±0.01^{bcdefgh}	46.1±0.14^{klm}
	C-17	10.5±0.03^{DEFGHIJ}	1.70±0.01^{Zabcdefghi}	12.36±0.04^{qrstuv}	12.3±0.35^{IJKLM}	27.9±0.35^{UVWXYZabcdefg}	20.2±0.28^{MNOPQRSTUVWX}	64.0±0.01^{mn}	41.2±0.28^{VWXYZab}	40.5±0.78^{YZ}
	C-18	11.6±0.01^{XZabcdefg}	1.71±0.01^{abcdefghij}	10.29±0.04^{NOPQRSTUVWXY}	16.5±0.71^{Zabcd}	23.8±0.71^{MNOPQRST}	22.1±0.14^{UVWXYZabcdef}	62.9±0.14^{klm}	42.1±0.14^{XYZabcd}	42.9±0.14^{cdef}
	C-19	10.6±0.01^{FGHIJK}	1.84±0.01^{rstuvw}	10.59±0.03^{QRSTUVWXYZab}	16.5±0.71^{Zabcd}	18.6±0.64^{HIJK}	24.0±0.01^{efghijklm}	56.0±1.41^{WXY}	54.6±0.07^{qrs}	36.2±0.28^{NOPQR}
	C-20	10.5±0.07^{DEFGHI}	1.65±0.01^{UVWXYZ}	10.86±0.03^{UVWXYZabcdefg}	11.0±0.01^{DEFGH}	28.5±0.71^{VWXYZabcdefgh}	18.2±0.42^{GHIJKLMN}	55.4±0.14^{UVWX}	35.6±0.14^{JKLMN}	36.1±0.14^{NOPQ}
	C-21	11.2±0.04^{PQRSTUVWY}	1.83±0.01^{qrstuv}	11.57±0.04^{cdefghijklmnopq}	13.0±0.71^{LMNOP}	26.0±0.28^{RSTUVWXYZa}	19.2±0.28^{JKLMNOPQR}	63.1±0.14^{lm}	37.1±0.14^{MNOPQ}	43.5±0.01^{efgh}
	C-22	10.5±0.02^{DEFGHI}	1.75±0.01^{ghijklmnop}	11.57±0.42^{cdefghijklmnopqr}	13.8±0.35^{OPQRS}	33.4±0.49^{lmnopq}	18.9±0.07^{IJKLMNOP}	62.3±0.28^{jkl}	36.3±0.35^{LMNO}	43.7±0.21^{fghi}
	C-23	10.7±0.09^{HIJKLM}	1.79±0.01^{mnopqrstu}	11.48±0.08^{bcdefghijklmnop}	9.8±0.35^{BC}	28.9±0.28^{YZabcdefghi}	21.5±0.01^{RSTUVWXYZabcd}	60.6±0.14^{ghi}	41.8±0.35^{XYZabc}	38.5±0.01^{VWX}
	C-24	12.6±0.05^{stuvwyxz}	1.46±0.01^{DEFGH}	10.76±0.09^{TUVWXYZabcd}	14.8±0.35^{STUVW}	13.5±0.46^{CD}	27.3±0.35^{opqrst}	32.0±1.41^{B}	48.5±0.01^{lm}	29.1±0.07^{D}
	C-25	11.7±0.03^{Zabcdefgh}	1.77±0.02^{jklmnop}	9.97±0.06^{LMNOPQRSTU}	14.3±0.03^{QRSTU}	13.2±0.06^{CD}	15.7±0.42^{BCDEF}	52.2±0.28^{PQ}	41.0±1.41^{UVWXYZab}	34.1±0.14^{KLM}
	C-26	11.1±0.08^{LMNOPQRST}	1.72±0.01^{bcdefghijk}	9.66±0.08^{IJKLMNOP}	13.3±0.05^{MNOPQ}	25.5±0.42^{QRSTUVWXY}	19.0±0.01^{IJKLMNOP}	53.0±0.07^{QR}	28.1±0.07^{AB}	41.6±0.07^{Zabc}
	C-27	11.7±0.04^{Zabcdefg}	1.56±0.01^{MNOPQR}	10.75±0.01^{TUVWXYZabcd}	12.8±0.07^{KLMNO}	27.1±0.07^{RSTUVWXYZabcd}	21.6±0.14^{TUVWXYZabcd}	70.5±0.07^{w}	35.4±0.14^{JKLMN}	45.6±0.14^{jkl}
	C-28	10.9±0.05^{JKLMNOPQ}	1.53±0.06^{JKLMNO}	11.76±0.18^{hijklmnopqr}	18.8±0.35^{e}	30.3±0.35^{cdefghijkl}	21.0±1.41^{OPRSTUVWXYZa}	74.1±0.07^{z}	43.1±0.14^{abcdef}	51.0±1.41^{o}
	C-29	13.0±0.02$^{Z\alpha\beta\gamma\delta}$	2.07±0.01$^{\chi}$	13.99±0.01^{x}	10.5±0.70^{CDEF}	39.0±0.28^{u}	11.3±0.28^{A}	58.6±0.14^{cdef}	38.6±0.14^{OPQRSTUV}	39.2±0.28^{XY}
	C-30	10.8±0.08^{IJKLMNO}	1.78±0.01^{klmnopq}	10.11±0.07^{MNOPQRSTUVW}	11.3±0.35^{EFGHI}	25.3±0.35^{PQRSTUVWXY}	16.8±0.25^{DEFGHI}	52.0±0.01^{PQ}	31.0±0.01^{DEFGH}	34.1±0.14^{KLM}
	C-31	10.6±0.07^{FGHIJK}	1.73±0.01^{defghijklm}	11.74±0.06^{fghijklmnopqr}	13.0±0.01^{LMNOP}	32.6±0.64^{jklmno}	17.0±0.01^{DEFGHIJ}	52.0±0.01^{PQ}	31.3±0.35^{DEFGH}	34.0±0.01^{KLM}
	C-32	13.0±0.03$^{Z\alpha\beta\gamma\delta}$	1.66±0.01^{VWXYZab}	10.50±0.08^{PQRSTUVWXYZa}	12.3±0.03^{IJKLM}	19.5±0.71^{HIJK}	25.5±0.07^{klmnopq}	66.6±0.07^{qrst}	46.1±0.14^{ghijkl}	23.5±0.01^{B}
	C-33	10.2±0.06^{ABCDE}	1.65±0.01^{UVWXYZ}	10.24±0.04^{NOPQRSTUVWX}	11.0±0.01^{DEFGH}	26.9±0.57^{RSTUVWXYZabcd}	15.1±0.14^{BCD}	54.7±0.28^{STUVW}	31.4±0.14^{DEFGH}	38.2±0.42^{UVWX}
	C-34	11.4±0.30^{STUVWYXZa}	1.66±0.04^{VWXYZab}	7.77±0.07^{ABC}	17.0±0.05^{bcd}	14.8±0.64^{CDEFG}	27.7±0.42^{rst}	35.6±0.14^{E}	100.9±7.28$^{\tau}$	32.5±0.01^{GHIJ}
	C-35	10.3±0.08^{BCDEFG}	1.85±0.02^{stuvw}	7.66±0.01^{AB}	11.8±0.35^{GHIJK}	11.5±0.71^{C}	20.5±0.07^{NOPQRSTUVWX}	37.0±0.01^{F}	63.0±1.41^{y}	30.7±0.21^{E}
	C-36	10.8±0.06^{IJKLMNO}	1.62±0.01R^{STUVWX}	9.66±0.40^{IJKLMNOP}	11.0±0.01^{DEFGH}	26.2±0.57^{RSTUVWXYZa}	19.8±0.35^{LMNOPQRSTU}	56.3±0.35^{XYZ}	36.0±1.41^{JKLMNO}	41.5±0.07^{Zab}
	C-37	11.1±0.07^{MNOPQRST}	1.87±0.03^{vwxy}	12.00±0.04^{mnopqrstu}	13.5±0.71^{NOPQR}	25.9±0.21^{RSTUVWXYZ}	20.0±0.01^{MNOPQRSTUV}	71.0±1.41^{wx}	36.1±0.07^{JKLMNO}	44.0±1.41^{fghi}
	C-38	11.1±0.04^{OPQRSTUVW}	1.45±0.03^{CDEFG}	10.62±0.05^{QRSTUVWXYZab}	12.8±0.35^{KLMNO}	26.5±0.71 ^{RSTUVWXYZab}	21.1±0.14^{PQRSTUVWXYZa}	55.4±0.21^{TUVWX}	52.1±0.14^{opq}	39.0±0.01^{WX}
	C-39	12.0±0.05^{efghijklmn}	1.73±0.01^{defghijklm}	10.65±0.03^{RSTUVWXYZab}	11.0±0.05^{DEFGH}	26.8±0.35 ^{RSTUVWXYZabcd}	26.6±0.14^{nopqrs}	69.0±1.41^{v}	43.5±0.01^{bcdefg}	46.1±0.14^{klm}
	C-40	11.6±0.1^{YXZabcde}	1.74±0.01^{efghijklmno}	11.15±0.06^{XYZabcdefghijklm}	17.5±0.07^{d}	30.4±0.42^{defghijklm}	16.5±0.01^{CDEFGH}	44.5±0.01^{LM}	66.2±0.28^{z}	32.2±0.28^{FGHI}
	C-41	11.3±0.25^{STUVWYXZ}	1.55±0.04^{KLMNOPQ}	10.03±0.06^{LMNOPQRSTUVW}	10.5±0.01^{CDEF}	19.4±0.07^{HIJK}	20.6±0.14^{OPQRSTUVWX}	50.5±0.07^{O}	56.4±0.21^{stu}	39.1±0.07^{WX}
	C-42	12.7±0.04$^{uvwyxz\alpha}$	1.88±0.03^{vwxyz}	10.64±0.11^{QRSTUVWXYZab}	11.0±0.10^{DEFGH}	26.5±0.35^{RSTUVWXYZab}	15.7±0.28^{BCDE}	57.0±0.41^{YZab}	36.1±0.07^{JKLMNO}	39.0±0.01^{WX}
	C-43	10.0±0.05^{ABCDE}	1.64±0.01^{UVWXYZ}	10.70±0.08^{RSTUVWXYZabc}	11.3±0.35^{EFGHI}	25.5±0.35^{QRSTUVWXY}	20.0±1.41^{MNOPQRSTUV}	58.0±1.41^{bcde}	44.3±0.35^{cdefghi}	43.6±0.14^{efghi}
	C-44	12.0±0.06^{hijklmno}	1.70±0.04^{Zabcdefgh}	10.50±0.25^{PQRSTUVWXYZa}	12.0±0.04^{HIJKL}	26.4±0.64^{RSTUVWXYZa}	17.2±0.28^{DEFGHIJK}	48.0±0.40^{N}	48.0±1.41^{jklm}	37.5±0.01^{QRSTUV}
	C-45	11.9±0.05^{defghijkl}	1.60±0.01^{QRSTUVW}	11.20±0.11^{YZabcdefghijklm}	11.8±0.35^{GHIJK}	26.7±0.21 ^{RSTUVWXYZabc}	25.1±0.14^{jklmno}	61.2±0.28^{hij}	40.5±0.07^{TUVWXYZa}	45.0±0.01^{ijk}
	C-46	11.2±1.06^{QRSTUVWYX}	1.69±0.01^{Zabcdef}	9.75±0.16^{JKLMNOPQ}	10.0±0.05^{BCD}	25.9±0.28 ^{RSTUVWXYZ}	69.3±1.13^{v}	55.1±0.14^{TUVWX}	41.0±1.41^{UVWXYZab}	27.0±1.41^{C}
	C-47	11.1±0.04^{MNOPQRSTU}	1.42±0.02^{BCDE}	10.57±0.11^{QRSTUVWXYZab}	15.8±0.35^{WXYZa}	23.3±0.78^{LMNOPQR}	22.0±1.41^{UVWXYZabcdef}	67.5±0.07^{rst}	49.0±1.41^{mn}	48.9±0.07^{n}
	C-48	12.3±0.11^{nopqrstuv}	1.68±0.01^{XYZabcd}	9.63±0.28^{IJKLMNOP}	14.8±0.05^{STUVW}	20.7±0.42^{IJKLM}	24.7±0.28^{ijklmn}	42.2±0.21^{HIJ}	37.5±0.07^{NOPQR}	35.0±0.07^{LMN}
	C-49	11.4±0.08^{STUVWYXZab}	1.72±0.01^{cdefghijkl}	11.39±0.10^{abcdefghijklmno}	14.8±0.06^{STUVW}	29.4±0.01^{Zabcdefghijk}	21.6±0.14^{STUVWXYZabcd}	65.5±0.01^{opq}	39.0±1.41^{PQRSTUVW}	40.8±0.35^{Za}
	C-50	11.7±0.01^{Zabcdefgh}	1.70±0.01^{Zabcdefghi}	10.98±0.01^{WXYZabcdefghijk}	10.8±0.35^{CDEFG}	24.0±0.28^{MNOPQRST}	21.0±1.41^{OPQRSTUVWXYZ}	62.0±0.07^{ijkl}	42.0±0.01^{XYZabcd}	42.3±0.35^{bcde}
	C-51	11.5±0.10^{VWYXZabc}	1.70±0.04^{Zabcdefghi}	10.66±0.30^{RSTUVWXYZab}	10.5±0.07^{CDEF}	24.8±0.35^{PQRSTUV}	21.4±0.21^{QRSTUVWXYZabc}	64.2±0.21^{mn}	28.5±0.07^{ABC}	41.5±0.01^{Zabc}
	C-52	12.2±0.01^{klmnopqrs}	1.45±0.02^{CDEFG}	7.82±0.07^{ABC}	10.3±0.35^{BCDE}	13.1±1.20^{CD}	16.7±0.21^{DEFGH}	34.3±0.35^{CD}	41.4±0.14^{WXYZab}	27.0±1.41^{C}

*Values are calculated as dry basis. **Values are calculated as 14% moisture content.Values in the same column with different superscripts indicate a statistically significant difference ($p<0.05$). AM: torque 15s before maximum torque, MT: maximum torque, PMT: peak maximum time, PM: torque 15s after maximum torque, GPU: GlutoPeak Unit

Supplementary Material

Table S1. Continue

Region	Sample	Moisture Content (%)	Ash content* (%)	Protein content* (%, N*5.7)	Zeleny sedimentation value** (mL)	Wet Gluten (%)	AM (GPU)	MT (GPU)	PMT (s)	PM (GPU)
CORUM	C-53	10.9±0.07^{KLMNOPQR}	1.62±0.01^{RSTUVWX}	10.74±0.14^{TUVWXYZabcd}	15.8±0.35^{WXYZa}	22.1±0.28^{KLMNOPQ}	20.8±0.35^{OPQRSTUVWXY}	56.5±0.01^{XYZa}	52.0±1.41^{op}	37.1±0.14^{PQRSTU}
	C-54	10.5±0.24^{DEFGHIJ}	1.81±0.01^{pqrstu}	12.45±0.01^{rstuv}	9.8±0.30^{BC}	33.9±0.42^{mnopqr}	20.0±1.41^{MNOPQRSTUV}	58.3±0.28^{bcdef}	34.2±0.28^{IJKL}	41.1±0.07^{Zab}
	C-55	11.2±0.03^{PQRSTUVWY}	1.56±0.01^{MNOPQRST}	10.10±0.01^{MNOPQRSTUVW}	11.8±0.35^{GHIJK}	24.4±0.64^{NOPQRSTU}	14.2±0.28^{B}	47.1±0.07^{N}	35.5±0.01^{JKLMN}	34.2±0.28^{KLM}
	C-56	10.7±0.13^{GHIJKL}	1.11±0.05^{A}	10.72±0.05^{STUVWXYZabc}	8.8±0.05^{A}	25.6±0.64^{QRSTUVWXY}	23.7±0.28^{defghijklm}	57.5±0.01^{Zabc}	39.8±0.28^{RSTUVWXY}	42.0±0.07^{abcd}
	C-57	12.2±0.18^{lmnopqrst}	1.37±0.01^{B}	8.18±0.06^{BCDEF}	13.3±0.05^{MNOPQ}	1.7±0.28^{A}	18.1±0.14^{GHIJKLM}	21.5±0.71^{A}	63.2±0.42^{y}	18.5±0.01^{A}
	C-58	11.0±0.04^{LMNOPQRST}	2.05±0.01$^{\tau\chi}$	11.69±0.05^{efghijklmnopqr}	17.5±0.07^{d}	33.4±0.57^{lmnopq}	18.7±0.28^{HIJKLMNO}	65.5±0.01^{opq}	44.9±0.21^{efghi}	44.1±0.07^{fghi}
	C-59	12.2±0.06^{jklmnopqr}	1.48±0.01^{FGHIJ}	12.09±0.15^{nopqrstuv}	16.8±0.05^{abcd}	28.9±0.92^{XYZabcdefghi}	19.2±0.28^{JKLMNOPQR}	64.1±0.14^{mn}	44.5±0.01^{defghi}	43.9±0.14^{fghi}
	C-60	12.2±0.13^{klmnopqrst}	1.59±0.01^{PQRSTU}	12.72±0.12^{tuvw}	16.3±0.05^{YZabc}	32.9±0.71^{klmnop}	23.5±0.01^{bcdefghijklm}	70.8±0.35^{wx}	42.3±0.28^{YZabcde}	49.1±0.14^{n}
	C-61	11.0±0.04^{LMNOPQRS}	1.60±0.02^{QRSTUV}	10.88±0.04^{UVWXYZabcdefghi}	12.5±0.70^{JKLMN}	26.6±0.07^{RSTUVWXYZabc}	16.0±1.41^{BCDEFG}	58.9±0.14^{cdef}	32.0±0.01^{EFGHI}	38.1±0.07^{TUVWX}

*Values are calculated as dry basis. **Values are calculated as 14% moisture content.Values in the same column with different superscripts indicate a statistically significant difference ($p<0.05$). AM: torque 15s before maximum torque, MT: maximum torque, PMT: peak maximum time, PM: torque 15s after maximum torque, GPU: GlutoPeak Unit
